# Gamma-Smooth Muscle Actin Expression Is Associated with Epithelial-Mesenchymal Transition and Stem-Like Properties in Hepatocellular Carcinoma

**DOI:** 10.1371/journal.pone.0130559

**Published:** 2015-06-25

**Authors:** Nassima Benzoubir, Charlotte Mussini, Charlène Lejamtel, Alexandre Dos Santos, Claire Guillaume, Christophe Desterke, Didier Samuel, Christian Bréchot, Marie-Françoise Bourgeade, Catherine Guettier

**Affiliations:** 1 Inserm, Unité 785, Villejuif, F-94800, France; 2 Univ Paris-Sud, UMR-S 785, Villejuif, F-94800, France; 3 AP-HP Hôpital Paul Brousse, Centre Hépatobiliaire, Villejuif, France; 4 SFR André Lwoff, Villejuif, F-94800, France; 5 Institut Pasteur, Paris, France; 6 AP-HP Hôpital Kremlin-Bicêtre, Service d’Anatomopathologie, Paris, France; 7 DHU Hepatinov, Villejuif, France; The University of Hong Kong, HONG KONG

## Abstract

**Background and Aims:**

The prognosis of hepatocellular carcinoma (HCC) is hampered by frequent tumour recurrence and metastases. Epithelial-Mesenchymal Transition (EMT) is now recognized as a key process in tumour invasion, metastasis and the generation of cancer initiating cells. The morphological identification of EMT in tumour samples from the expression of novel mesenchymal markers could provide relevant prognostic information and aid in understanding the metastatic process.

**Methods:**

The expression of Smooth Muscle Actins was studied using immunofluorescence and immunohistochemistry assays in cultured liver cells during an induced EMT process and in liver specimens from adult and paediatric HCC series.

**Results:**

We report here that in HCC cell lines treated with TGF-β and in HCC specimens, the expression of αSMA, a known mesenchymal marker of EMT, could never be detected. In addition, our *in vitro* studies identified the enteric form of SMA, γSMA, as being a marker of EMT. Moreover, this SMA isoform was expressed in 46% of 58 tumours from 42 adult HCC patients and in 90% of 16 tumours from 12 paediatric HCC patients. Interestingly, this expression was significantly correlated with poor tumour differentiation and progenitor cell features characterized by the expression of EpCAM and K19.

**Conclusion:**

Taken together, our results support the conclusion that γSMA expression in HCC is strongly correlated with the EMT process, HCC aggressiveness and the identification of cancer stem cells. This correlation suggests that γSMA represents a novel and powerful marker to predict HCC progression.

## Introduction

Hepatocellular carcinoma (HCC) is a major health problem in that it is the fifth most common cancer in the world and the third most frequent cause of cancer-related deaths. Most cases of HCC (80%) occur in livers that have become cirrhotic due to chronic Hepatitis B or C viral infection, alcohol abuse or obesity; all these conditions are characterized by long-standing hepatocyte damage and chronic inflammation leading to fibrosis [1]. Current chemotherapies are unable to exert a significant impact on patient survival. Although partial liver resection and liver transplantation have significantly improved survival with small tumours, the prognosis for HCC remains poor because of tumour invasiveness, frequent intrahepatic spread and extrahepatic metastases [2]. A clearer understanding of the molecular mechanisms underlying tumour invasiveness is therefore essential for the development of new therapies for HCC. It has been suggested that epithelial to mesenchymal transition (EMT) might be closely associated with the acquisition of aggressive traits by tumour cells, thus facilitating the early stages of metastasis and the subsequent dissemination of carcinoma cells [3] [4].

EMT is defined as a process during which epithelial cells lose their phenotypic characteristics and acquire mesenchymal cell features. Characteristic changes during EMT include the down-regulation of epithelial markers such as E-cadherin, and the up-regulation of mesenchymal markers such as vimentin and alpha smooth muscle actin (αSMA) [5]. More recently, EMT was linked to the emergence of cancer stem cells (CSC) [6] [7]. Indeed, it is now established that neoplastic epithelial cells re-enter the stem cell state through EMT. This has raised the intriguing possibility that the aggressiveness of carcinomas derives not only from the existing content of CSC but also from their proclivity to generate new CSC from non-CSC populations [8]. The correlation of carcinoma cell plasticity due to EMT with CSC properties may help to explain the role of CSC in the multistep progression of cancer. Indeed, oncogenic mutations that normally occur in differentiated cancer cells may involve CSC arising from EMT-induced de-differentiation, and these CSC with new oncogenic mutations may then contribute to the progression of cancer towards metastasis. The emergence of these CSC may largely contribute to the resistance of cancers to chemotherapies.

The molecular mechanisms underlying EMT development have been studied extensively *in vitro*, and for liver cells, primary cultures of hepatocytes or hepatoma cell lines are able to develop EMT under stimuli such as TGF-β. Any morphological analysis of carcinoma by pathologists rarely recognizes the mesenchymal features of tumour cells on tissue slides. Indeed, EMT is a dynamic and continuous event that may be difficult to characterize in tumours because it only involves few cells at a given time. In the context of HCC, several studies have demonstrated a correlation between an up-regulation of EMT inducers such as transcription factors, Snail or Twist, and tumour invasiveness [9]. Such a correlation has also been established between the loss of E-cadherin, an epithelial marker, and poorly differentiated HCC [10]. The morphological identification of EMT in tumour samples from the expression of mesenchymal markers could offer a relevant prognostic marker and also help to understand the metastatic process. In order to identify on tissue sections the small minority of cells undergoing EMT among the tumour cell population, an immunohistochemical technique is the most appropriate approach. In addition, formalin fixed paraffin-embedded tissue samples are available for every tumour.

At present the best-identified mesenchymal marker of EMT in tumour cells is αSMA. The human genome contains six functional actin genes that are expressed in various muscle and non-muscle tissues [11]. Two smooth muscle actins have been described, αSMA which is encoded by the *ACTA2* gene and γSMA encoded by the *ACTG2* gene. Although they differ in their sequence by only three amino acids, different studies have described αSMA as the predominant variant in vascular and respiratory smooth muscle, with γSMA being the predominant isoform in smooth muscle cells of the gastrointestinal and urogenital tracts. Because of their initially defined expression pattern, αSMA and γSMA are also referred as α-vascular and γ-enteric actins [12]. Although the role of γSMA is largely unknown, γSMA expression was able to functionally compensate for the lack of αSMA in myofibroblasts of αSMA knock-out mice [13].

We report here that the γSMA isoform could be considered as an EMT marker in HCC cell lines. Moreover, immunohistochemical analysis of a series of 58 tumours from adult patients and 16 tumours from paediatric patients revealed that γSMA, but not αSMA, was expressed in tumorous hepatocytes. The relevance of γSMA as an EMT marker for liver cells has been substantiated by *in vitro* data showing that this actin isoform becomes highly polymerized in hepatoma cell lines where EMT is stimulated by either TGF-β or over-expression of the Snail and Twist transcription factors. In view of the role of EMT in the emergence of CSC, the correlation between the expression of γSMA and progenitor markers (i.e. EpCAM or CK19) in HCC suggests that this actin isoform may represent a marker for non-CSC to CSC conversion. These inter-conversions are potentially important to the prognosis for HCC because CSC are more prone to dissemination and display resistance to anti-tumour therapies.

## Materials and Methods

### Ethics

Liver specimens were obtained from the Centre de Ressources Biologiques Paris Sud (CRB). All the samples collected by CRB are associated with a written consent of the patients. Samples are delivered after approval of the research project by the Scientific board of the CRB and thus satisfied requirements of the CRB ethics commitee. Access to this material was in agreement with French laws.

### Materials

Recombinant TGF-β1 was purchased from Abcys, Phalloidin was from InVitrogen.

### Vectors

The human cDNA clones pCMV6-Snail1 and pCMV6-Twist1 were obtained from Origen. The expression vector for pcDNA3.1_nV5 ACTG2 (V5-γSMA) was a kind gift from Dr Tuupanen.

### Cells

The human hepatoma cell line HuH7, and the LX-2 human stellate cell line, were maintained in Dulbecco Modified medium containing 10% foetal calf serum (FCS). HuH7 cells were transfected with the different vectors using XtremeGENE HP DNA reagent (Roche).

Primary tumour hepatocytes were isolated from patients undergoing liver surgery for HCC, after obtaining their written informed consent. The tissue samples were washed in Williams medium, cut into 2 mm^3^ fragments and incubated with Collagenase (Sigma Aldrich) (500 μg/ml, 2.4 mg/ml CaCl_2_ in HEPES buffer, pH 7.4) at 37°C for 15 min. The cells were then washed twice and plated in Williams medium supplemented with 10% foetal calf serum. After 4 hours, the serum-containing medium was removed and the cells were cultured in Williams medium supplemented with 1 mg/ml bovine serum albumin, 100 μg/ml streptomycin and 100 U/ml penicillin, and treated with 2 ng/ml TGF-β for 48h. They were then processed for immunofluorescence.

### Patients and tissue samples

Surgical liver specimens from 42 consecutive adult patients who underwent curative hepatic resection (n = 19) or liver transplantation (n = 23) for HCC at Hôpital Paul Brousse between January 1 and December 31, 2008, and 12 surgical specimens from paediatric patients who underwent hepatic resection (n = 5) or liver transplantation (n = 7) for HCC at Hôpital Bicêtre between 2006 and 2012, were obtained from (CRB Paris Sud), and analysed for the purposes of the study. The 58 HCC nodules identified in the 42 liver surgical specimens from adult patients, and the 16 HCC identified in the 12 surgical specimens from the paediatric patients were reviewed by two pathologists. The diagnosis of HCC was confirmed for each nodule using standard morphological criteria and HepPar1 and/or the immunohistochemical expression of glypican. The following histopathological features were systematically assessed: size, tumour capsule, satellite nodules, percentage necrosis, differentiation according to Edmondson’s grading, presence of cholangiolocellular and/or cholangiocellular component [14], macro and microvascular invasion.

Age, gender, underlying liver disease, type of surgery, and pre-and post-surgical treatments were recorded from the clinical charts.

As controls, 10 surgical liver specimens from normal livers, and 10 surgical liver specimens from cirrhotic livers were used to define γSMA staining in non-tumour liver tissues.

### Antibodies

Antibodies directed against HepPar1, glypican3, EpCAM, cytokeratin 19 (K19), αSMA, (clone 1A4), vimentin (Clone V9) and E-cadherin were obtained from Dakocytomation, Glostrup, Denmark. Anti-γSMA antibodies (Clone E184) were obtained from Epitomics. Anti-Snail, anti-Twist and anti-V5 antibodies were sourced from Abcam. Anti-p38 antibody came from SantaCruz Biotechnology, and anti-calcitonin antibody was from Diagnostic Biosystems.

### Western blotting

Cells were washed twice with PBS and lysed in RIPA buffer containing 0.5% SDS and Benzonase nuclease. Proteins were quantified with the Lowry assay and separated on SDS polyacrylamide gel, transferred on nitrocellulose membrane and blotted with different antibodies. Membranes were revealed using a chemioluminescence detection kit (ECL Plus, GE Healthcare) using a DCC camera (G Box Syngene).

### Immunofluorescence staining

Cells were washed with PBS and fixed with a 4% PFA solution at 4°C for 20 min and permeabilized with PFS (saponin gelatin in PBS) for 30 min at 37°C. Cells were then incubated with primary anti-αSMA, γSMA or V5 antibody, and then with secondary Alexa Fluor 488 conjugated anti-mouse or Alexa Fluor 594 conjugated anti-rabbit antibody (Molecular Probes). They were then stained with Hoechst and examined by fluorescence microscopy.

### Immunohistochemistry

Paraffin-embedded tissue sections were deparaffinized and antigenic restoration was achieved by heating in citrate buffer pH6. Immunostaining was performed on these tissue sections using a Bond Max automate based on a labeled streptavidin-biotin (LSAB) method.

All immunohistochemical staining results were scored by two observers. Discrepant results were reexamined and assigned after concordant opinion. For combined tumors with cholangiolocellular and/or cholangiocellular component, only the HCC component was analyzed.

HepPar1, glypican3, K19 cytoplasmic staining, E-cadherin and EpCAM membranous staining were scored as a percentage of positive tumor cells. Moreover, the staining intensity of tumor cells for E-cadherin was assessed in comparison with the non-tumor adjacent hepatocyte staining as overexpressed, isoexpressed or underexpressed. αSMA and γSMA staining was defined as membranous and/or cytoplasmic and scored as a percentage of positive tumor cells. Negative controls for γSMA staining were performed with a rabbit anti-calcitonin antibody. The threshold of positivity was defined as ≥10% for K19, EpCAM and γSMA.

### Statistics

Detection of the γSMA histological marker enabled the distinction of two groups of patients (positive and negative for this marker). Odds ratios for each clinical and biological criterion were calculated for each group of patients (γSMA-positive and γSMA-negative). A meta-analysis performed on all the clinical and biological criteria taken into account during the study allowed testing of the heterogeneity of the calculated odds ratio. A forest plot was drawn using the odds ratios and 95% confidence intervals for each criterion. The significance of the meta-analysis was retained if the p-value of the heterogeneity test was lower than 0.05 and if the random effects on the forest plot were well centred [15].

## Results

### γSMA is expressed in hepatocarcinoma cells undergoing EMT

**Fig 1:**
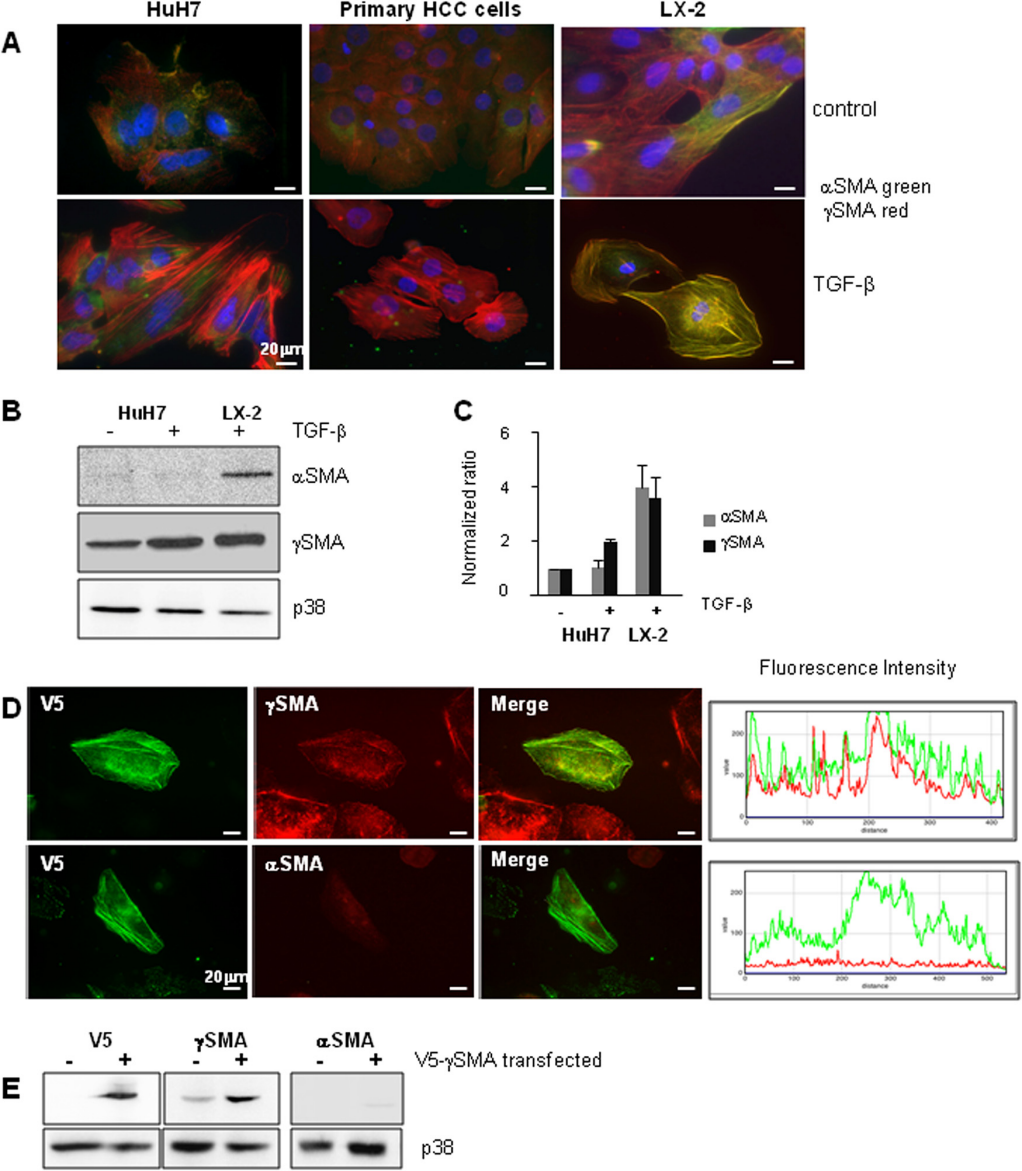
γSMA is expressed in TGF-β-induced EMT. (A) HuH7 cells and primary HCC tumour cells were treated with TGF-β for 48h and the expression of γSMA and αSMA was determined by immunofluorescence analysis. The images represent the merging of αSMA and γSMA staining. LX-2 stellate cells activated by TGF-β for 48h were used as a positive control for αSMA expression; one out of three representative experiments is shown. (B) HuH7 cells were treated with TGF-β for 48h and cell extracts were analysed by Western Blotting for the expression of αSMA and γSMA. TGF-β-treated LX-2 cells were used as positive controls for αSMA expression and p38 antibody was used as the control loading. (C) Densitometric analyses of the SMA/p38 ratio represent the mean +/- SD of three independent experiments. (D) HuH7 cells were transfected with a V5-tagged vector coding for γSMA. Cells were treated with TGF-β for 48h, and the expression of V5, γSMA and αSMA was determined by immunofluorescence analysis. Expression levels of V5 and γSMA or V5 and αSMA were determined by measuring fluorescence intensity on a linear section of the cell. One representative experiment is shown. (E) HuH7 cells were transfected or not with a V5-tagged vector coding for γSMA and cell extracts were analysed for the expression of V5, αSMA and γSMA.

Using an anti-SMA antibody, we had previously reported that SMA was up-regulated in HuH7 cells undergoing EMT after treatment with TGF-β [16]. Using specific antibodies directed against alpha or gamma SMA, the present study focused on the expression and polymerization of these two actin isoforms in hepatic cells undergoing EMT following treatment with TGF-β. As shown in Fig 1A, immunofluorescence staining revealed a strong polymerization of γSMA in TGF-β-treated HuH7 cells in conditions where αSMA was not detectable. The same results were obtained when primary HCC cells were treated with TGF-β; suggesting that the gamma isoform of SMA might represent a mesenchymal marker of liver tumour cells. Hepatic LX-2 stellate cells were used as positive controls for αSMA expression, and interestingly the expression of both SMAs was observed in these cells with strong labelling after activation by TGF-β. Furthermore, Western blot analysis demonstrated that αSMA was not detected in hepatic cells, whether they were or were not treated with TGF-β, while as expected, αSMA expression was easily detectable in LX-2 stellate cells after activation by TGF-β (Fig 1B and 1C).

The specificity of the anti-αSMA antibody used here is largely validated. However, few anti-γSMA antibodies are available. In order to verify that our anti γSMA antibody did indeed identify the γSMA isoform, we used a tagged-γSMA construct and studied the recognition of γSMA and αSMA antibodies in cells over-expressing the γSMA form. HuH7 cells were transfected with V5-tagged γSMA and analysed by immunofluorescence after 48h treatment with TGF-β. As shown in Fig 1D, a strict colocalisation was found when co-labelling with anti-V5 and anti-γSMA antibodies was performed, and strong polymerization in fibres was observed. By contrast, no labelling was observed with αSMA antibodies despite the high homology between these isoforms. In line with this, Western blot analyses of cell extracts from HuH7 cells transfected or not with V5-γSMA showed that an over-expression of V5-γSMA was recognized by anti-V5 and anti-γSMA antibodies but not by anti-αSMA antibodies (Fig 1E).

**Fig 2:**
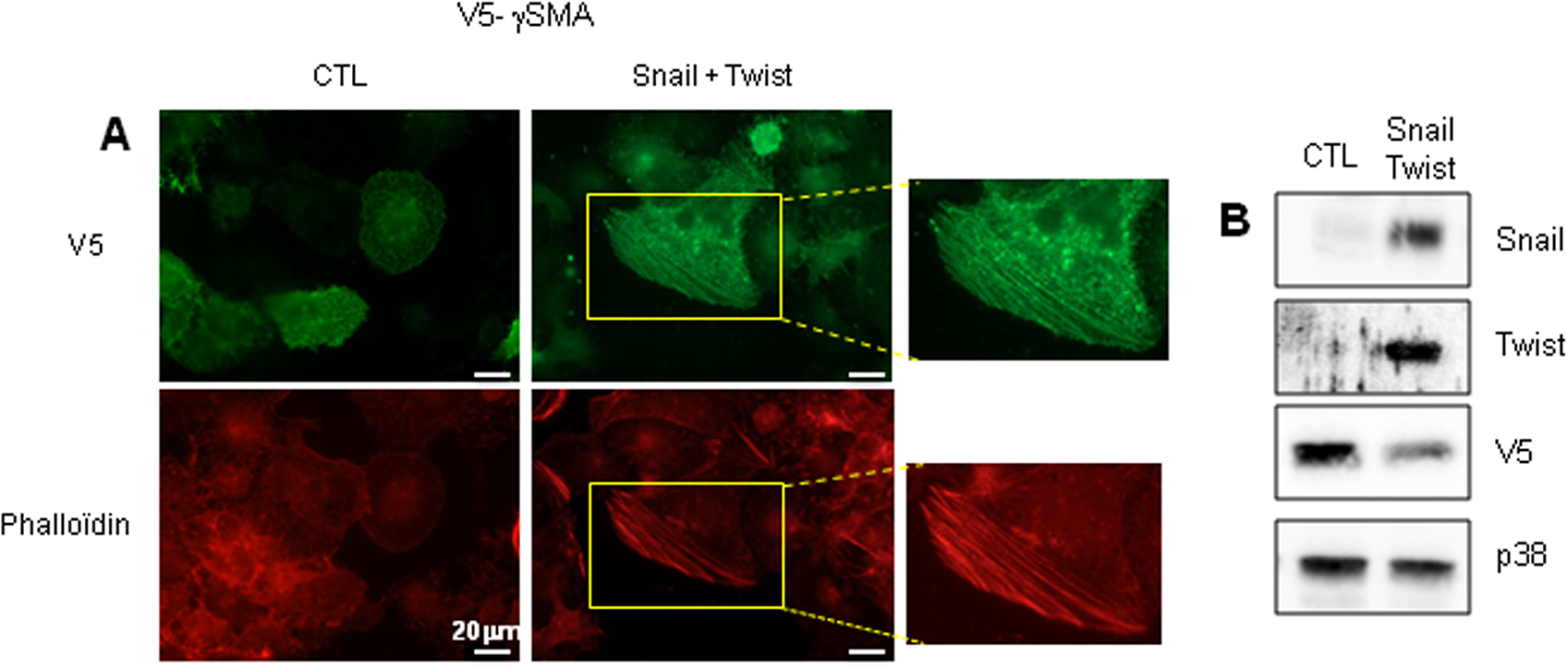
Snail and Twist expression induced γSMA polymerization. (A) HuH7 cells were transfected with a V5-tagged vector coding for γSMA alone (CTL) or together with vectors coding for Snail and Twist. The expression of V5 was determined by immunofluorescence analysis. Actin polymerization was revealed by Phalloidin staining. (B) The expression of V5, γSMA, Snail and Twist was analysed by Western blotting. p38 was used as a control loading.

Although EMT is regulated by an elaborate interplay of signalling pathways, the full molecular reprogramming that occurs during EMT is mainly orchestrated by three major groups of transcription factors: the ZEB, Snail and Twist families. The exogenous over-expression of Twist1 increases the invasive and metastatic abilities of human cancer cells by promoting the down-regulation of E-cadherin and the induction of EMT. Likewise, the over-expression of Snail and Twist in hepatoma cell lines promotes EMT and the acquisition of an invasive phenotype [17]. Thus in order to gain further insight into the involvement of γSMA in EMT, we looked for γSMA polymerization in HuH7 cells over-expressing Twist1 and Snail1. For this purpose, HuH7 cells were transfected with a V5-tagged γSMA vector, with or without Twist and Snail vectors. Immunofluorescence staining was performed 48h after transfection. The polymerization of V5-tagged-γSMA was present in cells expressing Snail and Twist (Fig 2A). This correlated with the findings of staining with phalloidin, a compound known to detect actin polymerization. No such polymerization of γSMA could be observed in cells expressing only V5-tagged γSMA (Fig 2A). Western blotting analyses revealed the expression of Twist, Snail and V5 in the same experiment (Fig 2B).

Taken together, these results strongly suggest that γSMA is polymerized in hepatic cells having developed EMT induced by TGF-β or the over-expression of transcription factors such as Snail and Twist.

### γSMA is expressed in human HCC

*In vitro*, EMT has emerged as a pivotal event in development of the invasive and metastatic potentials of cancer progression. However, although *in vivo* cancer cells undergo a de-differentiation process, EMT is still difficult to detect due to the tumour heterogeneity that is one of the hallmarks of HCC, and the transitory nature of EMT or EMT-like process. Our *in vitro* data prompted us to investigate whether γSMA expression might represent a marker for EMT that could be easily processed in tissue sections and used in routine clinical practice.

**Fig 3:**
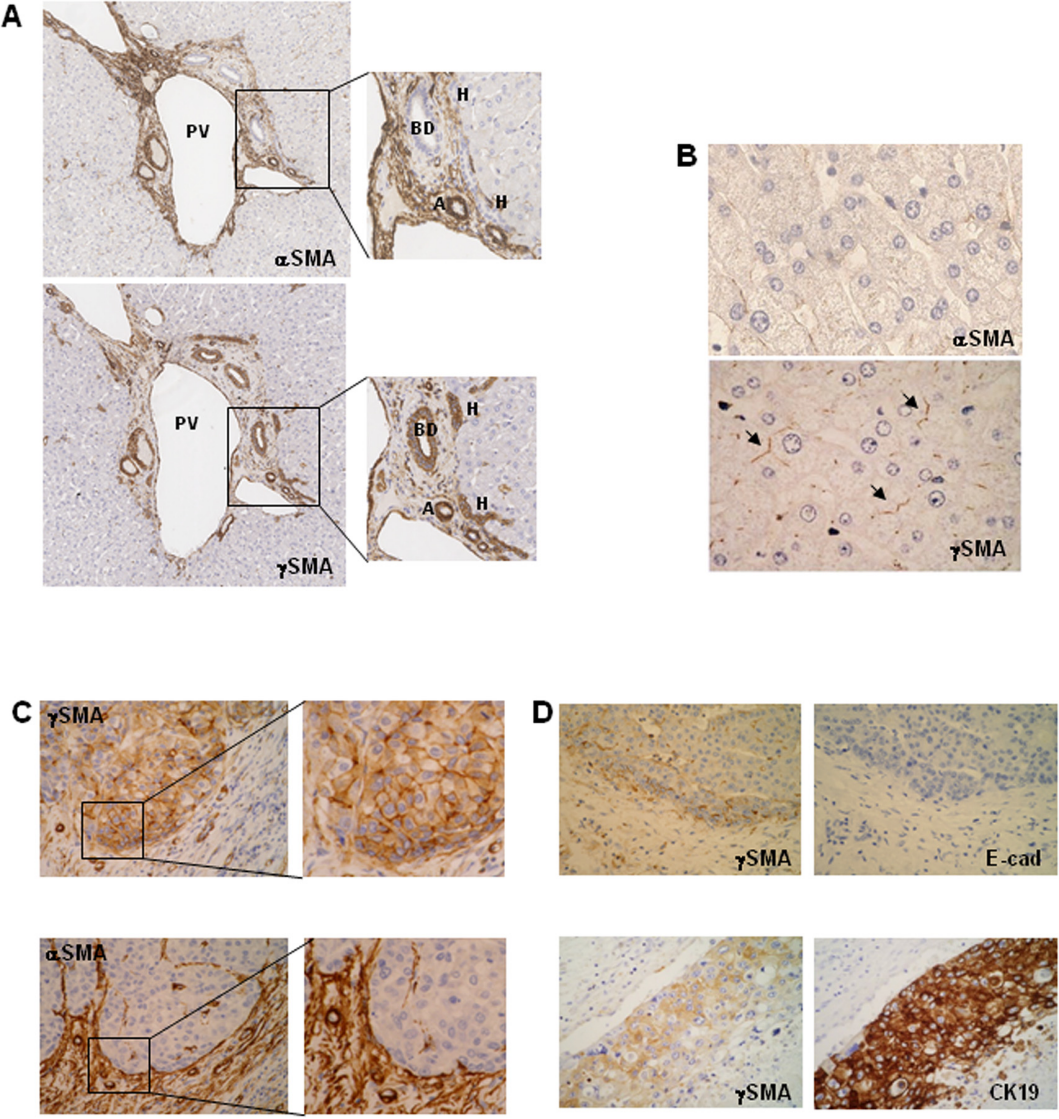
Immunochemical analyses of αSMA and γSMA expression in normal and HCC livers. (A) Serial sections of normal livers were immunochemically stained with anti-αSMA or anti-γSMA antibodies. BD: bile duct, A: artery, H: canal of Hering, PV: Portal Vein. x50. (B) Sections of normal livers were immunochemically stained with anti-αSMA or anti-γSMA antibodies. (**→**: canaliculi) x400. (C) (D) HCC nodules were immunochemically stained with different antibodies. Serial sections stained with γSMAand αSMA; x160 and x400 (C). γSMA and E-Cadherin (E-cad); γSMA and CK19 (D). x160.

We first of all determined the expression of α and γSMA in ten normal livers. Both actin isoforms were expressed in the arteries, but their expression differed in other liver structures. Indeed, αSMA expression was mainly found in the non-parenchymal compartment. Interestingly, the canals of Hering were strongly labelled with γSMA antibodies and always negative with αSMA antibodies, suggesting that only the γSMA form is expressed in hepatic progenitor cells. The bile ducts were also positive for SMA (Fig 3A). Neither αSMA nor γSMA could be detected in hepatocyte cytoplasm and membranes, although a discrete labelling for γSMA was observed in the canaliculi (Fig 3B).

The same results were obtained in ten cirrhotic livers with different aetiologies. γSMA was inconsistently expressed at the canalicular pole of hepatocytes. In HCC-affected cirrhotic livers, γSMA was not expressed in the cytoplasm of non-tumour hepatocytes, except focally in one case. Ductules within the fibrous bands were consistently positive, as were the bile ducts.

**Fig 4:**
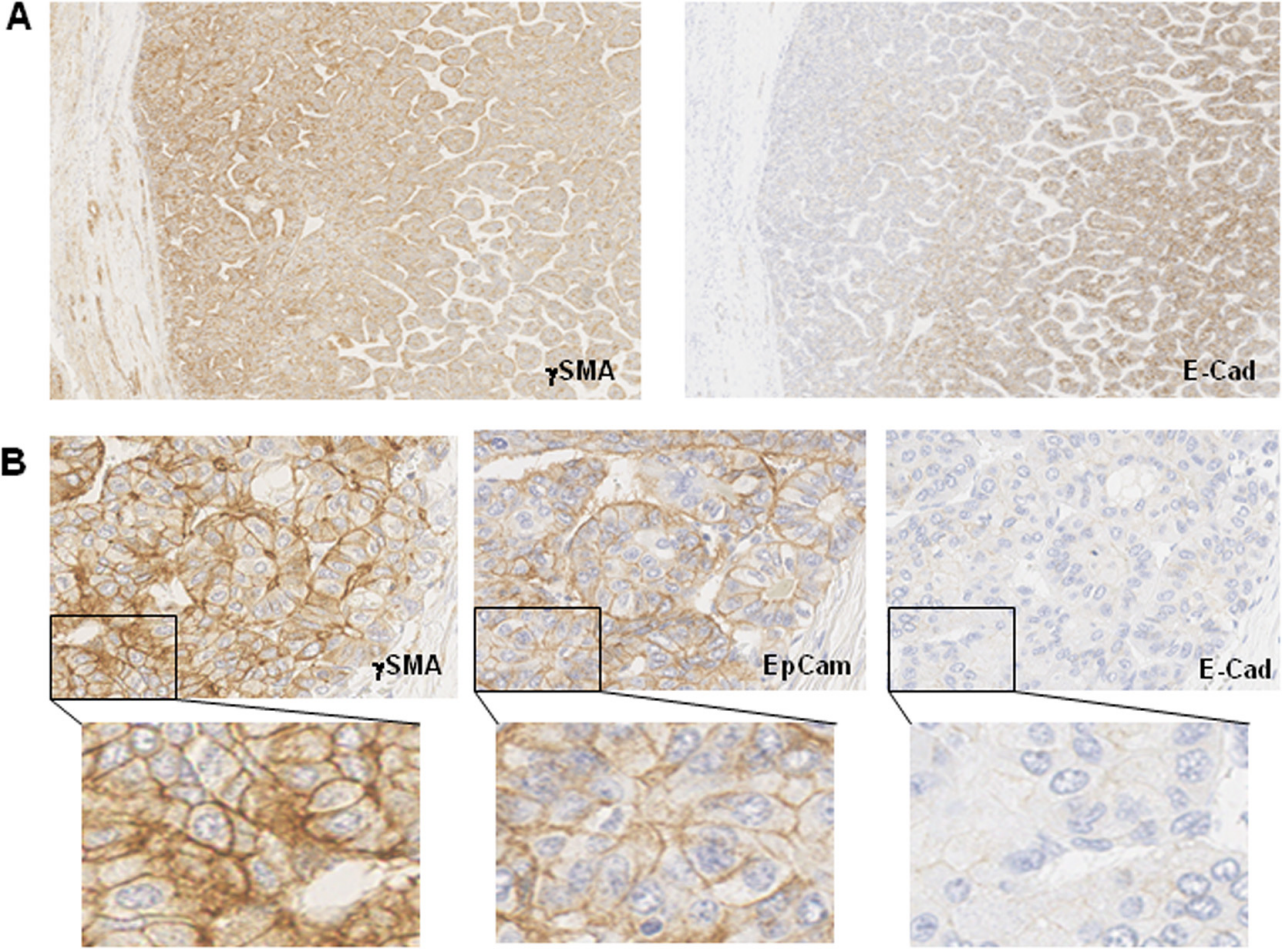
Serial sections of paediatric HCC samples were immunochemically stained with different antibodies. (A) γSMA, and E-cad. x50. (B) γSMA, E-cad and EpCAM. x400 and x1000.

In the context of HCC, a total of 58 nodules corresponding to 42 patients were examined for γSMA expression. It was never possible to detect αSMA in tumour hepatocytes, whereas the expression of this actin form was easily detectable in the stromal compartment (Fig 3C). By contrast, γSMA was detected in 46% of the tumours (Fig 3C). Staining was observed only in tumor hepatocytes with membranous and/or cytoplasmic pattern (Fig 3C high magnification). γSMA expression was heterogeneous and frequently observed at the invasive front of the tumour, but rarely throughout the nodule. In the panel of paediatric HCC samples, γSMA expression was observed in 15 out of 16 nodules, and here again its positivity was mainly observed at the edge of the nodules (Fig 4A).

**Fig 5:**
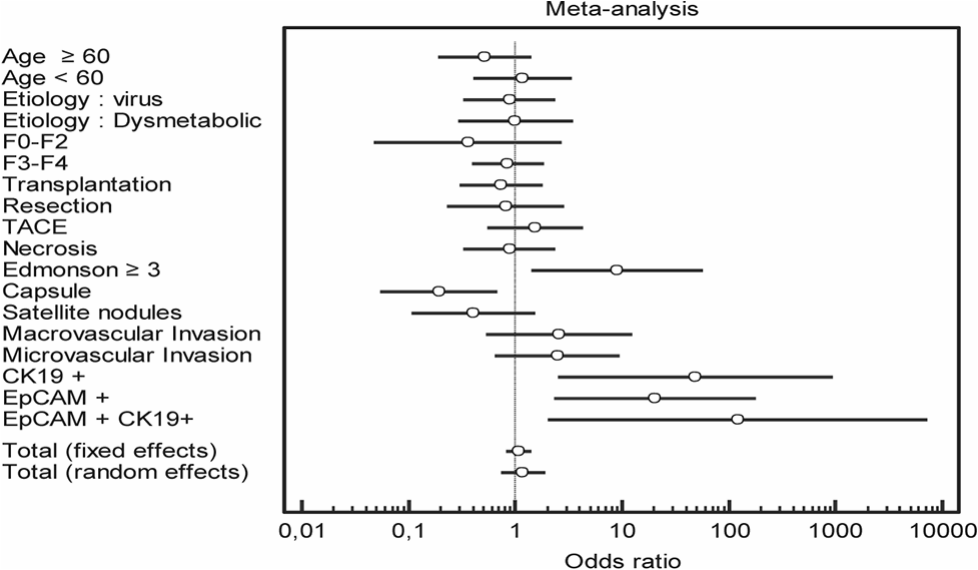
Odds ratio meta-analyses of data from 58 HCC nodules. Meta-analyses were performed on clinical and biological criteria.

Meta-analyses of these 58 HCC nodules from adult patients indicated that γSMA expression was not correlated to age, type of surgery (liver transplantation or resection), tumour necrosis or neoadjuvant TACE treatment (Table 1 and Fig 5). By contrast, in the context of adult HCC, γSMA expression was significantly associated with a poor degree of tumour differentiation. In line with this finding, the majority of paediatric HCC cases were classified as Edmondson 3.

**Table 1:** Correlation between γSMA expression, clinicopathological parameters and progenitor markers in HCC.

	Total	γSMA positive	Odds ratio	95% Confidence Interval
Total nodules	**58**	**27 (46.6%)**		
Age ≥ 60	31	13 (42%)	0.522	0.190 to 1.431
Age < 60	27	14 (51.8%)	1.16	0.399 to 3.373
Etiology :Viruses	31	15 (48.4%)	0.869	0.325 to 2.380
Etiology :Dysmetabolic	20	10 (50%)	1	0.289 to 3.454
F0-F2	8	3 (37.5%)	0.36	0.0476 to 2.725
F3-F4	50	24 (48%)	0.852	0.389 to 1.867
Transplantation	39	18 (46.2%)	0.735	0.302 to 1.790
Resection	19	9 (47.4%)	0.81	0.227 to 2.895
TACE	29	16 (55.2%)	1.515	0.538 to 4.264
Necrosis	31	15 (48.3%)	0.879	0.325 to 2.380
Edmonson ≥ 3	12	9 (75%)	9	1.418 to 57.119
Capsule	23	7 (30%)	0.191	0.0545 to 0.672
Satellite nodules	18	7 (38.9%)	0.405	0.106 to 1.547
Macrovascular Invasion	13	8 (61.5%)	2.56	0.527 to 12.431
Microvascular Invasion	18	11 (61.1%)	2.469	0.646 to 9.432
CK19 +	8	7 (87.5%)	49	2.531 to 948.671
EpCAM +	11	9 (81.8%)	20.25	2.319 to 176.800
EpCAM + CK19+	5	5 (100%)	121	2.017 to 7259.723

In order to correlate γSMA expression with a known marker of EMT, we studied the expression of E-cadherin, an epithelial marker, which is lost during EMT. Although γSMA expression could be focally associated with a complete loss of E-cadherin expression (Fig 3C), the extreme heterogeneity of E-cadherin staining within the same tumour hampered the demonstration of a firm correlation between a loss of E-cadherin and γSMA expression. However, in paediatric HCC, complete E-cadherin loss was observed in 13 out of 16 nodules, and E-cadherin expression was very weak in the others (Table 2 and Fig 4B). Thus a strong and significant association between γSMA expression and a loss of E-cadherin could be established, at least in the context of paediatric HCC.

**Table 2:** Correlation between γSMA, E-cadherin and progenitor marker expression in paediatric HCC.

	Total	γSMA positive	Odds ratio	95% Confidence Interval
Total nodules	**16**	**15 (93.7%)**		
E-cadherin negative	13	12 (92.3%)	144	8.043 to 2578.222
CK19 +	5	5 (100%)	121	2.017 to 7259.723
EpCAM +	14	14 (100%)	841	15.605 to 45324.593
EpCAM + CK19+	5	5 (100%)	121	2.017 to 7259.723

### Correlation between γSMA expression and stem cell markers

It is now well established that CSC can arise from tumour cells that have achieved EMT. Because of the strong expression of γSMA in the canals of Hering, we decided to determine whether a correlation exists between γSMA and the expression of stem cell markers in HCC. Interestingly, 7 out of the 8 HCC expressing CK19 were positive for γSMA staining. Furthermore, 9 out of the 11 HCC expressing EpCAM were also γSMA positive. Five CHC co-expressed CK19 and EpCAM, and all of them were positive for γSMA expression. Meta-analyses of the data indicated that γSMA expression and progenitor markers were statistically correlated (Table 1 and Fig 5). These data therefore suggest that γSMA may be expressed in cancerous cells displaying the hallmarks of progenitor cells. It has been reported elsewhere that transarterial chemoembolisation (TACE) treatment may be associated with the emergence of CSC [18]. It is noteworthy that γSMA positivity was not correlated with previous TACE treatment.

With respect to paediatric HCC, 14 out of 16 tumors concomitantly expressed γSMA and EpCAM. Among them, five nodules also expressed CK19 (Table 2). This result obtained in paediatric HCC therefore strongly support the hypothesis that γSMA may represent a marker of cancer stem cells or progenitor-like cells.

## Discussion

Emerging data strongly suggest that like in other cancers, EMT may play an important role in tumour progression and the emergence of cancer stem cells in a context of hepatocellular carcinoma (HCC).

It has been shown that *in vitro* primary cultures of hepatocytes or hepatoma cell lines are able to develop EMT under stimuli such as TGF-β or the over-expression of Twist or Snail transcription factors [16] [19]. Our study demonstrated that TGF-β-induced EMT resulted in γSMA expression and organization in stress fibres in HuH7 cells. The use of a tagged-γSMA vector allowed us to show that the antibody used in this study recognized the γSMA isoform. Moreover, the over-expression of Snail and Twist, two transcription factors necessary for EMT progression, was sufficient to induce γSMA polymerization. These data strongly support the notion that γSMA represents an EMT marker in liver cancer cell lines.

To our knowledge, γSMA has never before been reported as an EMT marker. However, in many studies, phalloidin was used to detect polymerized actin during the EMT process, although it does not distinguish between different polymerized actins. It is therefore possible that γSMA expression is not restricted to hepatoma cells and may also be increased and polymerized in other cell lines undergoing EMT. Interestingly, it has been reported that TGF-β induced transcriptional activation of the γSMA gene. Indeed, increased steady-state levels of γSMA mRNA and the induced production and cytoskeletal polymerization of γSMA protein were observed during TGF-β-induced mesenchymal to myofibroblast differentiation [20].

Variants of γSMA have been implicated in familial visceral myopathy, a rare inheritable disease characterized by the impaired contraction of visceral smooth muscle cells and reduced bowel motility [21]. However, few data are available regarding a role for γSMA in cancer cells: It has been reported that *ACTG2* encoding for γSMA is one of the two most up-regulated genes in highly aggressive osteosarcoma cell lines when compared to non-aggressive cell lines [22]. Furthermore, γSMA mRNAs were shown to be up-regulated in cultured primary HCC cells, and particularly in cells with an aggressive phenotype [23]. These correlations between γSMA expression and cell aggressiveness or cellular motility are in agreement with our data and strongly support the notion that γSMA could be a marker for the invasive phenotype of cancer cells.

These *in vitro* results prompted us to look for γSMA expression in human HCC tissue sections in order to clarify the EMT process *in situ*. To the best of our knowledge, this is the first report of γSMA immunohistochemistry on human liver tissue. The positivity of the canals of Hering in normal livers, and of the ductular reaction in cirrhotic livers, strongly suggests that γSMA is a marker of liver progenitor cells. The expression of γSMA in normal or cirrhotic hepatocytes were restricted to canaliculi, indicating that γSMA, as previously described in several organs [12], is not involved in liver fibrosis. Immunohistochemistry on HCC in adult patients confirmed the expression of γSMA in 46% of the tumours with membranous and/or cytoplasmic staining of tumour cells, whereas αSMA was only expressed in stromal cells. These different intrahepatic localizations are in favour of complementary roles for these two SMA isoforms. The positivity of γSMA was only focal, and more frequently observed at the edge of the tumour. Strikingly, γSMA was expressed in the tumour cells of all but one of the paediatric HCC patients.

Until now, the *in situ* identification of EMT in HCC using mesenchymal markers was largely unsuccessful and produced contradictory results. It was mainly based on αSMA and vimentin immunostaining. Several papers have reported the expression of αSMA in HCC tissues, but only in cancer associated-fibroblasts and myofibroblasts of the stromal compartment and not in tumorous hepatocytes [24] [25] [26]. However, in a recent paper, αSMA expression was detected at low levels by immunohistochemistry in HCC tumour cells [27]. Many anti-SMA antibodies recognize both and γSMA, and because no precise indication was given concerning the anti-SMA antibodies used in that study, it is difficult to ascertain whether it was αSMA rather than γSMA that was actually expressed in these tumour cells.

The second mesenchymal marker widely employed to characterize EMT is vimentin. *In vitro*, vimentin expression in normal primary hepatocytes and HCC cell lines can be correlated with an EMT process [16]. However, in our cohort, we were never able to detect vimentin expression in HCC nodules. In a previous paper, we reported vimentin expression in intrahepatic cholangiocarcinoma using immunohistochemistry on tissue microarrays (TMA), whereas the 19 HCC included in these TMA were all negative [28]. Thus the lack of vimentin detection in our present HCC cohort was not related to the antibodies used. In contradiction to our findings, vimentin expression in HCC tumour cells has been reported by different authors working on Asiatic cohorts [29] [30] [10]. These discordant results might be explained by the different aetiologies of European and Asian HCC. This discrepancy therefore warrants further investigation and might be clarified by comparing the expression of γSMA and vimentin in Asian cohorts.

As well as the over-expression of mesenchymal markers, EMT can also be characterized by a down-regulation of epithelial markers such as E-cadherin in HCC cells. However, in our study, E-cadherin staining appeared to be quite heterogeneous, even in non-tumour liver, so we were unable to demonstrate a statistically significant correlation between E-cadherin loss and γSMA expression at the level of whole tumours, even though this correlation could be observed focally. In paediatric HCC, E-cadherin was almost constantly down-regulated in association with γSMA expression.

Taken together, therefore, our results suggest that γSMA could be a robust and reliable *in situ* mesenchymal marker for an *in vivo* EMT process in the context of HCC.

γSMA expression in HCC was significantly associated with known histologically pronostic factors, such as poor tumour differentiation (Edmondson ≥ 3) and lack of tumour capsule formation. Moreover, although they did not meet the criteria for statistical significance, our data suggest that a correlation between γSMA expression and vascular invasion might also exist. Because most of the patients in our study benefited from liver transplantation, which totally modifies the natural history of HCC, it was not possible to determine a link between γSMA expression, tumour recurrence and overall survival.

Interestingly, γSMA expression in HCC tumour cells was strongly associated with stem/progenitor cell markers, namely CK19 and EpCAM. This result is in agreement with γSMA expression in the canals of Hering and ductules, which are supposed to be the locations of adult liver progenitor cells. HCC with stemness-related marker expression is a recently proposed subtype of HCC in which a fraction of tumour cells (>5%) expresses stem/progenitor cell markers such as K19, CD133, c-kit, and EpCAM [31]. This subtype is associated with the expression of EMT markers and characterized by a poorer prognosis than HCC without stemness or progenitor markers.

Further evidence of the association between γSMA expression and stemness features was obtained by studying the paediatric HCC cohort. Indeed, these HCC differ from those in adults, with a nearly constant expression of stem/progenitor cell markers such as EpCAM, which was expressed in 14 out of the 16 tumours in our paediatric study. This result is in total agreement with a recent paper by Zen et al. who reported the expression of EpCAM in 25 out of 26 HCC nodules from a cohort of 12 children [32]. For these authors, EpCAM expression in childhood HCC may be attributable to the immaturity of neoplastic cells. In our series, the co-expression of γSMA and EpCAM, together with the down-regulation of E-cadherin, is consistent with the paradigm that cancer stem-like cells might arise from cancer cells via an EMT process.

It would be of interest to examine the γSMA status of patients in the setting of other gastrointestinal cancers in order to determine whether expression of this protein is restricted to HCC or might represent a more general marker of CSC in endoderm-derived cancers.

Determining predictive biomarkers for HCC prognosis remains a challenge. The identification of γSMA as a potential mesenchymal marker of EMT, as well as EMT-induced stemness, constitutes an appreciable advance in predicting HCC progression and could be exploited for prognostic benefit.
